# Cryoneurolysis of Intercostal Nerves for Postherpetic Neuralgia: A Case Report

**DOI:** 10.7759/cureus.70557

**Published:** 2024-09-30

**Authors:** Arun Kalava, Ronaldo Kassie, Ellie Borick

**Affiliations:** 1 Anesthesiology, University of Central Florida College of Medicine, Orlando, USA; 2 Pain Management, TampaPainMD, Tampa, USA; 3 Medicine, University of Central Florida College of Medicine, Orlando, USA

**Keywords:** chronic pain management, cryoneurolysis, herpes zoster bilateralis symmetricus, intercostal neuralgia, postherpetic neuralgia

## Abstract

Cryoneurolysis is a procedure that has been shown to be efficacious in managing pain in various settings. Multiple studies have demonstrated the benefit of cryoneurolysis; however, the literature on managing postherpetic neuralgia with cryoneurolysis is scarce. The current treatment options available include transdermal lidocaine patches, capsaicin, non-steroidal anti-inflammatory drugs, nerve blocks, antidepressants, anticonvulsants, and opioids. Pain-relieving treatments such as opioids can have long-term detrimental effects on a patient or be ineffective for long-term pain relief, so there is cause to explore alternative treatment options. Here, we present a case of ultrasound-guided cryoneurolysis of the intercostal nerves for the management of postherpetic neuralgia after herpes zoster bilateralis symmetricus. After several treatment options were unsuccessful in alleviating this patient's pain, cryoneurolysis of the intercostal nerves proved to be a very effective pain management tool.

## Introduction

Herpes zoster, also known as shingles, presents as a painful, dermatomal rash that appears when a latent infection of the varicella-zoster virus reactivates [1]. Bilateral herpes zoster is a rare clinical manifestation of herpes zoster infection in which the typical blistering rash is present, but both sides of the trunk are affected. In herpes zoster duplex bilateralis, there is involvement of two separate dermatomes, whereas in herpes zoster bilateralis symmetricus, the rash crosses the midline in a continuous presentation, without adhering to the single dermatome [2]. This is an extremely rare presentation of herpes zoster, only occurring in 0.1% of cases [3].

Postherpetic neuralgia is defined as a chronic neuropathic pain condition that persists for three or more months after an episode of herpes zoster [4]. This condition results in severe patient suffering and reduced quality of life and is costly both to the individual and to the healthcare system to treat [5]. Patients of advanced age and those with compromised immunity are more likely to develop herpes zoster and subsequent postherpetic neuralgia [6]. Treatments such as transdermal lidocaine patches, capsaicin, non-steroidal anti-inflammatory drugs (NSAIDs), nerve blocks, antidepressants, anticonvulsants, and opioids are all common treatment options for managing pain caused by this condition [5]. The literature is scarce thus far on the effectiveness of cryoneurolysis in managing postherpetic neuralgia, despite it being a prominent cause of chronic neuropathic pain. The primary advantage of cryoneurolysis for patients is that it is an effective minimally invasive treatment option that minimizes or eliminates the need for oral and topical medications.

Cryoneurolysis is a procedure that uses extremely cold temperatures applied directly onto nerves to disrupt the conduction of pain signals from the peripheral to the central nervous system and reduce patient discomfort [7]. Cryoneurolysis is a minimally invasive procedure that should be considered to manage pain, in comparison to pharmacological treatments such as opioids that can have long-term effects on patient well-being [8]. Studies exploring the effectiveness of cryoneurolysis have shown it to be a safe and effective treatment for chest pain, with positive results also demonstrated for the treatment of chronic refractory peripheral mononeuropathies [9,10].

The procedure is centered around an “ice ball” on the tip of a probe that creates temperatures of -60°C or lower. When applied to an affected nerve, this leads to interruption of nerve conduction and Wallerian degeneration. The nerve structures remain intact after the procedure, allowing for eventual nerve regeneration [11]. The use of cold temperatures for therapeutic purposes is well-documented in history with Hippocrates being the first to use snow and ice applied to wounds. One of the first studies on cryoneurolysis in 1976 by Lloyd et al. found that cryoneurolysis was superior in preventing neuralgia and neuritis that often followed neuroablation with phenol, alcohol, or surgical lesions [12]. Based on previous literature, cryoneurolysis could be further explored as a potential treatment option for patients with chronic neuropathic pain.

## Case presentation

A 58-year-old male with a past medical history of herpes zoster, type 2 diabetes mellitus for 16 years, and right carpal tunnel syndrome presented with shingles pain in the lower posterior thoracic and abdominal area. Pain was reported to be 9/10 on the visual analog scale (VAS) with a constant electrical and burning quality, radiating across his entire back, and worsened when sitting still. The patient’s history of herpes zoster began with lesions on the right thoracic and abdominal areas and after resolution, recurred six months later with bilateral lesions. On physical exam, healed zoster lesions were noted at the T7-T9 dermatomes of the posterior thoracic wall bilaterally. Previous treatments that attempted to reduce the pain were unsuccessful and included gabapentin and transdermal lidocaine patches.

**Figure 1:**
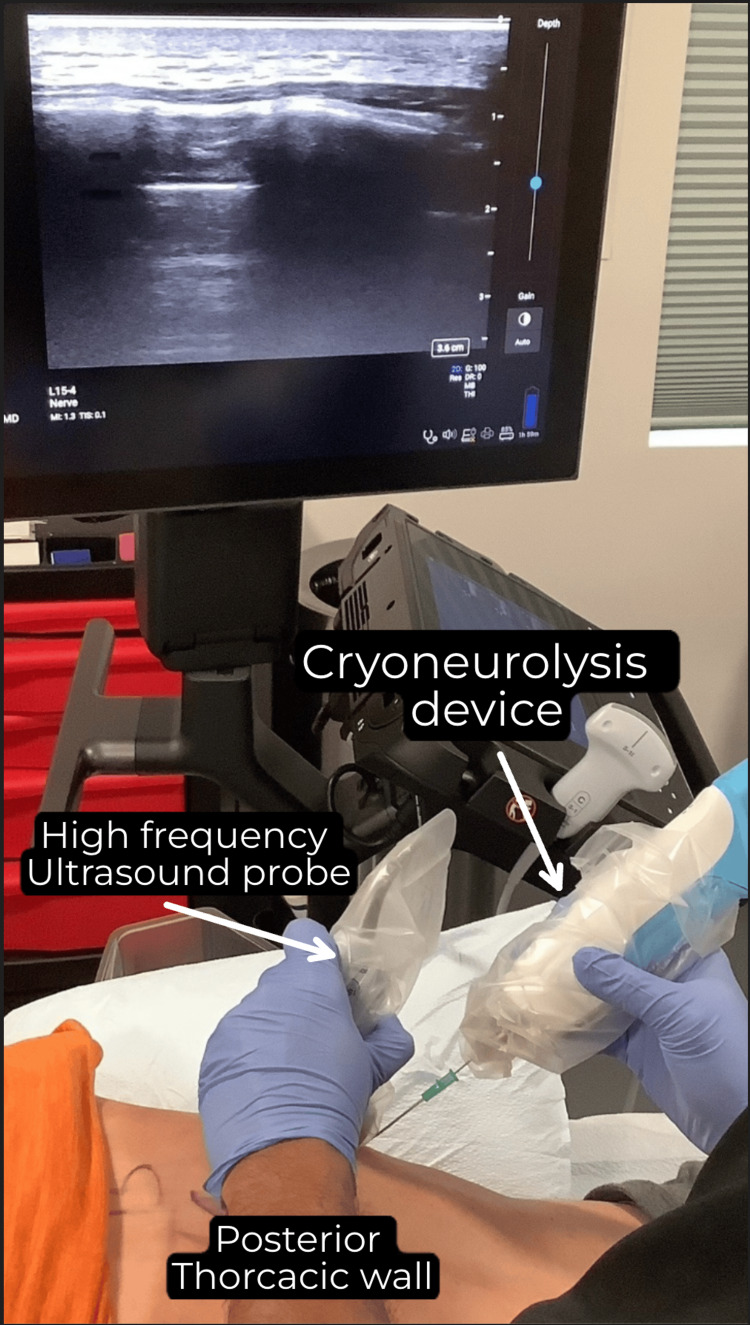
Cryoneurolysis procedure being performed.

The patient experienced a greater than 50% reduction in pain symptoms after five sessions of localized cryotherapy (four to six minutes, at -70°C) and class IV laser treatments. However, still, in a great deal of pain, the patient developed a diffuse rash from pinching his abdomen to try and alleviate the pain and pointed out continual pain in the regions of the T6, T7, and T8 dermatomes on the left and the T6 dermatome on the right. Initially, intercostal nerve blocks of the left T5, T6, T7, and T8 and the right T6 and T9 intercostal nerves were performed. This was done under ultrasound guidance using a 22-gauge Tuohy needle to inject 3 ml of 0.5% ropivacaine perineurally. Steroids were not used in the nerve block due to the patient's history of type 2 diabetes mellitus. Cryoneurolysis of the left T5 and T6 and the right T9 intercostal nerves was subsequently performed based on the patient’s response to the diagnostic intercostal nerve blocks (Figure 1).

**Figure 2:**
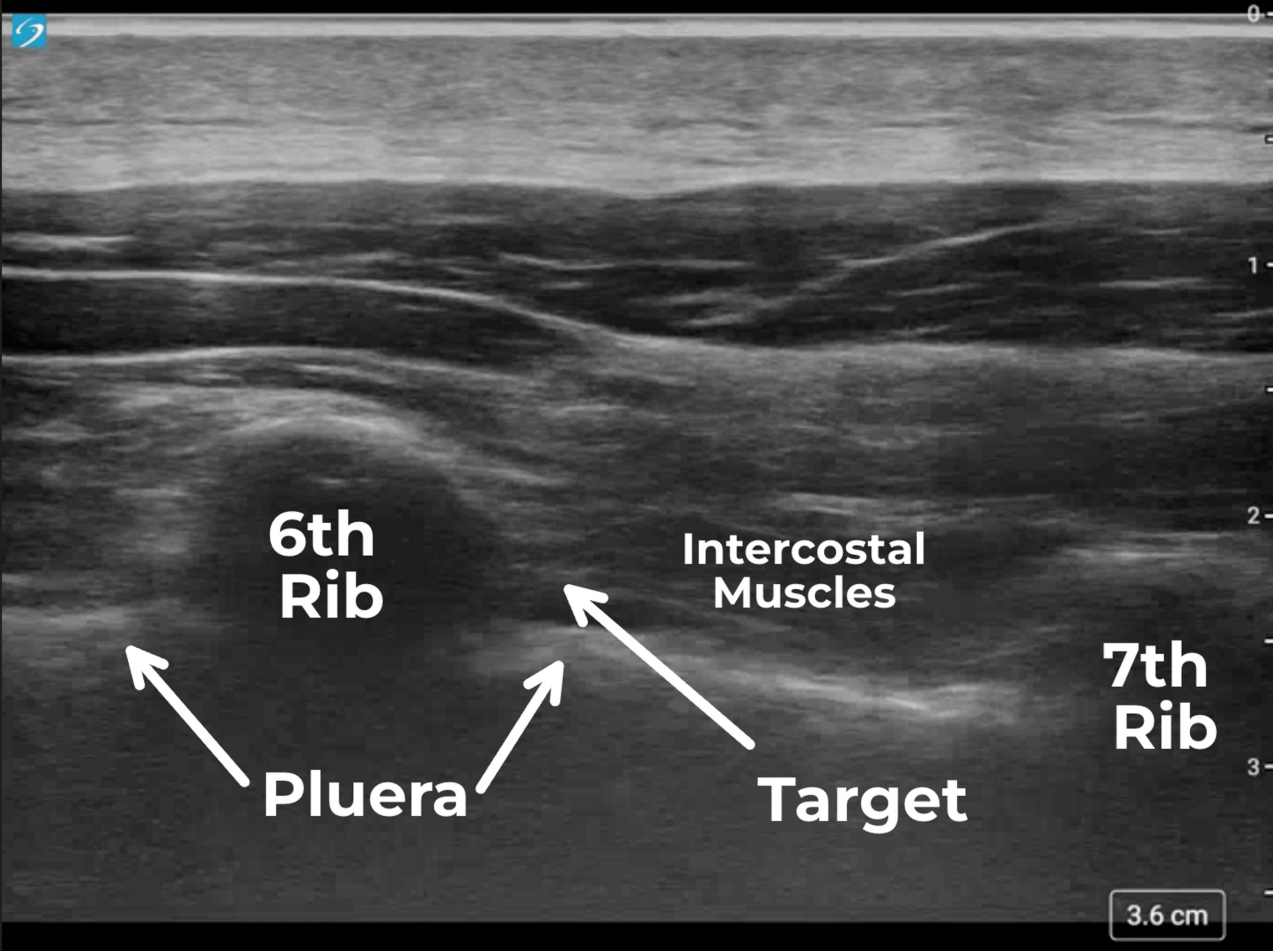
Ultrasound image of the 6th intercostal nerve being targeted for cryoneurolysis.

With the patient in a prone position, the area was sterilized with BD ChloraPrep containing 2% chlorhexidine gluconate (CHG) and 70% isopropyl alcohol (IPA). He was given a mixture of nitrous oxide and oxygen (50:50) to relieve procedural anxiety. A high-frequency (15-4Mhz) ultrasound probe, covered with a sterile sheath was placed in the anatomical location where the intercostal nerve is located. Once the target area was identified, 2 mL of 1% lidocaine was injected and a skin wheal was raised. An 18-gauge angiocatheter was inserted through the skin wheal. Using a sterile technique, a 20-gauge Pacira (Pacira Biosciences Inc., Parsippany, NJ) Iovera cryoneurolysis needle (Smart Tip 2190) was advanced through the angiocatheter with a tip positioned at the inferior aspect of the rib in between the inner and innermost intercostal muscle layers (Figure 2). Cryoneurolysis was subsequently performed at -88°C for four cycles of 106 seconds. Each cycle included one second of pre-warming time, 60 seconds of cooling cycle, and 45 seconds of post-cooling. The needle was then withdrawn, and the injection site was cleansed and covered with a bandage.

The procedure was well-tolerated by the patient and no complications or signs of hematoma were observed. He was subsequently discharged in a stable condition. The patient experienced complete resolution of pain on the posterior thoracic wall after cryoneurolysis; however, pain in the abdomen persisted. Follow-up with the patient’s wife via telephone one month after the procedure revealed that the patient had left the country to seek medical treatment in Kuwait. The patient’s wife reported that he still had pain in his abdomen, but no pain in his back. After two months post-procedure, the patient's wife reported that he was still in Kuwait and that she was not sure when he would be returning to the US. She stated that he was complaining of pain in the left abdomen alone and was managing the pain with capsaicin patches at that time.

## Discussion

This case highlights the suitability of cryoneurolysis in the management of refractory postherpetic neuralgia. The procedure was able to completely relieve the patient’s shingles pain in his posterior thoracic area, with sustained pain relief at one month post procedure. However, the patient still experienced pain in his abdominal region. This can be attributed to the fact that cryoneurolysis was only applied to three intercostal nerves (left T5 and T6 and right T9). We plan on targeting further intercostal nerves based on the patient’s readiness to continue treatment secondary to financial constraints. In discussing the role of cryoneurolysis here, it should be noted that other studies have recently yielded similar results in patients with both postoperative and chronic refractory pain.

Yoon et al. performed a prospective study between July 2011 and July 2013 with 22 patients being treated for refractory peripheral neuropathic pain using cryoneurolysis of various nerves. Mean pain decreased from 8.3 ± 1.9 before intervention to 2.3 ± 2.5 at one month, 3.2 ± 2.5 at three months, 4.7 ± 2.7 at six months, and 5.1 ± 3.7 at 12 months after intervention. There was a statistically significant change between pre- and post-treatment pain scores at each time interval (P < 0.5). Of note, 11 of the 22 patients underwent repeat cryotherapy treatments within 12 months of the initial procedure [13].

A systematic review was performed by Cha et al. in 2021 highlighting the efficacy of intercostal cryoneurolysis as an analgesic adjunct after chest wall surgeries or trauma. This review focused on 23 studies between 1974 and 2020, encompassing 575 individuals divided into groups based on the chest wall surgery performed. In summary, cryoneurolysis proved to be an effective adjunct to analgesia, reducing narcotics use and improving patient quality of life, all while having very few complications [14].

While both articles demonstrated the effectiveness of cryoneurolysis in treating chronic refractory pain, neither article included its use in the treatment of postherpetic neuralgia. Weber et al. (2019) published a case report in which cryoneurolysis was used to treat postherpetic neuralgia in a patient who presented with pain in the left arm, left back, and flank by targeting the left intercostobrachial nerve. This resulted in a greater than 50% decrease in pain severity, one-month post procedure, after which the patient required no further follow-up [15].

The differences in procedures throughout cases should be noted as well. Weber et al. utilized a three-pronged cryoablation tip probe to provide multiple cycles of cryotherapy lasting 60 seconds at a temperature of -88°C. In comparison, our study utilized the Pacira Iovera Smart Tip 2190 needle at the same temperature, for a total of four cycles, with each cycle consisting of one second of pre-warming time, 60 seconds within the cooling cycle, and 45 seconds of post-cooling. This was based on the manufacturer’s guidelines. Literature on the appropriate duration of time for exposing the nerve to this temperature varies but it is recommended to perform a series of two to three‐minute freezes with 30 seconds of defrosting between each cycle to allow subsequent freezes to increase the size of the freeze zone [7,15].

As with most procedures, there are some complications associated with cryoneurolysis. These include those common to interventional procedures such as bleeding, infection, damage to surrounding structures, and failure of the procedure. The use of ultrasound and nerve stimulation greatly improves accuracy and thus ensures that the correct nerve is being targeted and also reduces the risk of damage to neighboring structures. When targeting superficial nerves, skin damage can arise leading to conditions such as alopecia and issues with pigmentation. To prevent this, saline can be injected under the skin to elevate it [16].

Lastly, the patient’s comorbidities should be highlighted as to how they impacted the disease process. As mentioned above, advancing age, immunocompetence, and chronic diseases are all risk factors for herpes zoster infection, which would affect this patient. Lai et al. performed a meta-analysis in 2021 on the incidence of herpes zoster in patients with diabetes. They found that the overall risk of developing herpes zoster was significantly higher in patients with diabetes mellitus when compared to those with no diabetes mellitus (incidence rate ratio = 1.60, 95% confidence interval = 1.33-1.93) [17].

Compounding this is the cohort study of Wen et al. in 2023 on the impact of type 1 versus type 2 diabetes on developing herpes zoster and postherpetic neuralgia. Not only did they find that both type 1 and type 2 diabetes mellitus had a strong effect on the risk of developing postherpetic neuralgia as compared with non-diabetic individuals, but they also discovered that patients with both type 1 and type 2 diabetes had a 1.45-fold higher risk of developing postherpetic neuralgia than those without diabetes (hazard ratio = 1.45, 95% confidence interval = 1.28-1.65 and hazard ratio = 1.45, 95% confidence interval = 1.37-1.52, respectively). In addition, their study showed that diabetic patients in the 40-59 age group, of which this patient is in, had a two-fold increased risk of postherpetic neuralgia compared with non-diabetic individuals [18].

## Conclusions

We believe that this is the first report of the use of cryoneurolysis of intercostal nerves to treat postherpetic neuralgia from herpes zoster bilateralis symmetricus. Our patient experienced effective pain relief in the posterior thoracic region, with no observed complications from the procedure. Further studies would be valuable on the long-term effects of cryoneurolysis for postherpetic neuralgia.
