# Correction: Cryoneurolysis of Intercostal Nerves for Postherpetic Neuralgia: A Case Report

**DOI:** 10.7759/cureus.c297

**Published:** 2025-09-16

**Authors:** Arun Kalava, Ronaldo Kassie, Ellie Borick

**Affiliations:** 1 Anesthesiology, University of Central Florida College of Medicine, Orlando, USA; 2 Pain Management, TampaPainMD, Tampa, USA; 3 Medicine, University of Central Florida College of Medicine, Orlando, USA

The Ethics Statement and Conflict of Interest Disclosures section has been updated to disclose the following:

**Financial relationships:** Arun Kalava and Ronaldo Kassie declare(s) employment from TampaPainMD. TampaPainMD promotes and provides cryoneurolysis as a treatment.

